# Viewpoint on the Intersection Among Health Information, Misinformation, and Generative AI Technologies

**DOI:** 10.2196/69474

**Published:** 2025-09-15

**Authors:** António Bandeira, Luis Henrique Gonçalves, Felix Holl, Juliet Ugbedeojo Shaibu, Mariana Laranjo Gonçalves, Ronan Payinda, Sagun Paudel, Alessandro Berionni, Young WFPHA, Tina D Purnat, Tim Mackey

**Affiliations:** 1 Unidade Local de Saúde Santo António, Unidade de Saúde Pública, Polo Gondomar Porto Portugal; 2 Bloomberg School of Public Health Johns Hopkins University Baltimore, MD United States; 3 Fundação Oswaldo Cruz Rio de Janeiro Brazil; 4 DigiHealth Institute, Neu-Ulm University of Applied Sciences Neu-Ulm Germany; 5 Leibniz Science Campus Digital Public Health Bremen Germany; 6 Technical Advice Connect Abuja Nigeria; 7 Global Health Policy Taskforce World Federation of Public Health Associations Geneva Switzerland; 8 STARS Brazil Initiative (Students and Trainees Advocating for Resource Stewardship) Londrina Brazil; 9 University of Auckland Auckland New Zealand; 10 Public Health Initiative Kathmandu Nepal; 11 World Federation of Public Health Associations Geneva Switzerland; 12 T.H. Chan School of Public Health Harvard University Cambridge, MA United States; 13 Global Health Equity and Digital Technology Working Group World Federation of Public Health Associations Geneva Switzerland; 14 Global Health Program, Department of Anthropology UC San Diego La Jolla, CA United States

**Keywords:** generative artificial intelligence, infodemics, public health, Health Information, Misinformation

## Abstract

In recent years, artificial intelligence (AI) has seen rapid advancements, with innovations such as large language models and generative AI evolving at a rapid pace. While this progress offers tremendous opportunities, it also presents risks, particularly in the creation, consumption, and amplification of information and its impact on population health and health program delivery. Thoughtful approaches are necessary to navigate the consequences of advances in AI for different health care professionals and patient populations and from a policy and governance perspective. Through a collaboration between the World Federation of Public Health Associations working groups, this Viewpoint article brings together perspectives, concerns, and aspirations from young adult professionals across 5 continents and from diverse backgrounds to explore the future of public health and AI in the context of the changing health information environment. Our discussion is divided into 2 parts, specifically examining aspects of disinformation and AI, and also the role of public health and medical professionals in a growing AI-driven health information ecosystem. This Viewpoint concludes with 5 key recommendations on how to potentially address issues such as information and disinformation overload; misinformation propagation; and resultant changes in health practices, research, ethics, and the need for robust policies that can dynamically address current and future challenges.

## Introduction

### Position Statement of the Authors

The World Federation of Public Health Associations (WFPHA) serves as a crucial global network uniting public health professionals and organizations committed to improving health outcomes and well-being worldwide. As a leading voice in global health, the WFPHA promotes public health advocacy, influences policy, and advances professional education across diverse regions. Within the WFPHA, the Young Working Group for the WFPHA (Young WFPHA) acts as a collection of medical and health professionals focused on empowering the next generation of public health leaders. Dedicated to fostering leadership, expanding career opportunities, and driving innovation, the Young WFPHA facilitates global collaboration among emerging professionals committed to advancing health and well-being. The group’s vision is to ensure that young professionals are integral to building equitable and high-performing public health systems. The Young WFPHA, consistent with the mission of the WFPHA, is dedicated to equipping emerging public health professionals with the requisite skills and networks to address contemporary public health challenges, including in the context of new and emerging technologies. The Young WFPHA is also active in collaboration with various stakeholders and implements its mission through active engagement in prominent international health forums, including the World Health Assembly. In 2023, the Young WFPHA group conducted a detailed survey aimed at identifying the challenges encountered by early-career public health professionals. The findings identified major obstacles and concerns about the ability to navigate the expanding role of generative artificial intelligence (GenAI) in public health and health care design, delivery, and practice. This next generation of artificial intelligence (AI) tools presents both opportunities and challenges, requiring professionals to adapt and innovate in response to this rapidly evolving landscape. In response, the Young WFPHA partnered with the WFPHA Working Group on Global Health Equity and Digital Technology that focuses on research, education, training, and capacity building to ensure that digital technologies include health equity principles in their design, validation, implementation, and assessment. In this Viewpoint article, the 2 working groups bring together and elevate the diverse perspectives, concerns, and aspirations of young adult public health and medical professionals across 5 continents to explore the future of public health and AI in the context of the changing health information environment. GenAI can be defined as follows [1]:

Generative AI is artificial intelligence (AI) that can create original content, such as text, images, video, audio or software code, in response to a user’s prompt or request.

### The Emerging Role of Generative AI

The area of AI, which is broad in scope and generally comprises technologies and a field of science that focuses on building computers and machines that can reason, learn, and act in a way that normally requires human intelligence, has seen significant advancements with the introduction of large language models (LLMs) and GenAI applications that are becoming increasingly accessible worldwide. GenAI is aggressively being incorporated by Microsoft, Google, and Apple across their products and services—concomitantly, Meta launched the largest open-source LLM, Llama 3.1. Subsequently, GenAI is being integrated into many aspects of daily life, from content creation (text, images, and music) to productivity tools (automation of tasks, customer service, and coding), data analysis (insights, reports, and trend predictions), and agentic AI models (chatbot agents) transforming our daily lives. Furthermore, GenAI has been shown to be promising in incorporating technically complex knowledge, as exemplified by OpenAI’s ChatGPT’s 98% score on a US Medical Licensing Examination Step 3 mock exam [2].

On the other hand, GenAI is, in essence, modes that generate output based on the patterns of content and types of language in the datasets that were used for its training. Therefore, the quality and accuracy of the model’s outputs depend on the format, strategy, language, and knowledge base used in their training processes—when users use expert domain vocabulary, apply domain frameworks, and leverage domain context, they obtain better LLM outputs [3]. In specialized knowledge domains such as medicine, the implementation of LLMs in workflows is still nascent and will require improved evaluation approaches, consideration of prompt engineering, and understanding of human-computer interaction with the systems that incorporate GenAI, among other considerations specific to ensuring patient safety and improved health outcomes [3-6].

On the regulatory front, the G7 AI Principles and Code of Conduct have been established to guide the responsible and safe global use of AI. The European Union (EU) led the way with its comprehensive AI Act, positioning itself between China’s strict regulatory controls and the more innovation-friendly, self-regulatory approaches of the United States and United Kingdom. In the United States, the “Safe, Secure, and Trustworthy Development and Use of Artificial Intelligence” executive order originally set the framework for regulatory approaches complemented by state-level and industry-specific regulations but has since been revoked by the new administration through an executive order in an effort to remove barriers to AI development [7-10]. Together, these emerging data and AI governance frameworks will be important to assess in the context of their implications for applications in medicine and public health.

The GenAI transformation will arguably impact all levels of society. McKinsey & Company [11] estimates that GenAI could add US $4.4 trillion annually to the global economy, although there are concerns about potential loss of employment across multiple industrial sectors. However, less understood is how GenAI could impact health information systems and broader information ecosystems already at significant risk from a trust and integrity standpoint following the global COVID-19 pandemic that was accompanied by an equally complex “infodemic.” Tackling the challenges associated with infodemics during crises will be critical, particularly in the context of how infodemics in the age of polycrises can be mitigated or exacerbated by established or emerging technologies. We must also consider the broader challenge of the information ecosystems and how diverse types of information—unclear, outdated, conflicting, and low-quality health information; misinformation; and disinformation (defined later in this Viewpoint)—impact health information seeking, understanding, and use, as well as health behaviors.

Infodemic refers to an overabundance of information, accurate or not, that occurs during a mass acute health event such as an outbreak, epidemic, or event of mass importance. It can lead to confusion, risky behaviors, and mistrust in health authorities [12].

Outside of the health emergency context, the work and impact of public health systems and health professionals is influenced by the structures and dynamics of the information ecosystem. The *information environment* refers to the dynamic set of *ecosystems* of all the communication channels, platforms, actors, narratives, and interactions that influence how individuals receive, process, and use and act upon information. With the digitization of information exchange and the ubiquitousness of information consumption, these rapidly changing information ecosystems are straining the capacity of public health agencies and programs to provide credible and accurate health information to clinicians, patients, and the public [13,14].

LLMs, GenAI, and other emerging AI technologies and computational systems (eg, quantum computing) present both challenges and opportunities for public health but are rarely designed or implemented with the priorities of public health outcomes, health, and well-being in mind or adequate governance of potential for “dual use” (ie, they could be directly misapplied for threats with potential consequences for public health and safety) [15]. Accordingly, a balanced approach to adopting the tools supported by AI advancements, ensuring that these are both innovative and responsible, is necessary and should be informed from diverse perspectives [16].

To address this complex topic, this Viewpoint article gathers perspectives from young health professionals who represent stakeholders who are crucial in shaping the future of how the public health community assesses, responds to, and introduces into practice these technology changes. Specifically, this Viewpoint will explore the specific opportunities and risks posed by GenAI in the health and information sectors through a collaborative consensus-making process and then conclude with a discussion of essential steps in education, training, research, innovation, policy, and ethics to better ensure the consideration of responsible AI advancements that benefit public health.

## Approach Description

An initial draft outline of the manuscript concept was developed, followed by an open call for contributors based on the outlined topics. This call was disseminated among members of the Young WFPHA, leading to the purposeful selection of authors to ensure diverse representation in terms of geography and professional backgrounds. The participating contributors and coauthors came from Portugal, the United States, Nepal, Nigeria, New Zealand, Italy, Germany, and Brazil, representing diverse fields such as medicine, public health, medical informatics, and social psychology.

A collaborative approach toward generating consensus on main findings was used, with biweekly meetings where authors shared insights, proposed improvements, and agreed on new goals. For the 5 questions in part 2, each participating responding author independently and anonymously drafted and submitted their responses to the lead and first author. The latter then synthesized these responses into unified answers, which were then reviewed and refined by all authors until consensus was reached.

For the remainder of the manuscript, while all authors were encouraged to contribute to each subsection, each author was assigned the responsibility of compiling and integrating the content for their respective sections.

## Part 1: The Dynamics of Information Ecosystems in Health Through the Critical Lens of AI Innovation

In the first part of our Viewpoint article, we assess the threats and opportunities presented by GenAI tools and applications, specifically concerning disinformation overload, AI-generated misinformation, and societal impacts.

### Disinformation

*Disinformation* is deliberately promoted false information and can include hoaxes, conspiracies, and propaganda. Spreading health disinformation often exploits people’s vulnerabilities for profit or political or ideological influence and inconsistencies in accuracy, relevancy, and context of the information. This can be driven by harmful commercial industries, fraudsters, or profiteers in sectors such as the tobacco, alcohol, food, wellness, and health supplement industries. Some actors spread disinformation to gain influence and monetize their audiences, advancing political or ideological agendas. For example, antivaccination groups use strategies such as donations, membership fees, advertising, and merchandise sales to fund their activities [17]. In addition, geopolitical actors use disinformation to weaken political opponents. Attacks on health systems through propaganda or cyberattacks undermine both the quality of health care and public trust in institutions, eroding people’s sense of safety and social cohesion [18].

In today’s digital age, people are bombarded with vast amounts of information daily through many channels and devices, leading to *information overload*—a state in which the sheer volume of information becomes overwhelming for people, making it difficult to process and make well-informed decisions. To manage this complexity, the brain uses *cognitive shortcuts* (or heuristics)—quick mental strategies that allow individuals to make judgments without needing to analyze all details [17-20].

Both information overload and cognitive shortcuts contribute to analysis paralysis—when people feel overwhelmed and avoid engaging with information critically. Political, economic, or cultural agents can exploit this unbalanced information environment to advance their agendas with limited transparency, taking advantage of trust erosion and belief confirmation bias—where individuals prioritize information that confirms preexisting views and dismiss contradictory evidence, therefore making it harder to reach people with credible, accurate information [19-23].

In the *modern information environment*, human attention is treated as a scarce and valuable resource, which is sometimes called the *attention economy*. Platforms, content creators, and media outlets compete to capture the attention of their users, shaping how information is produced, distributed, and consumed to influence their users’ behaviors and keep them on their platforms as long as possible to derive advertising revenue [24-31].

As public health strongly relies on the acceptance and adoption of evidence-based health behaviors by the broader population, dynamics arising from information overload–related phenomena can be particularly harmful—these dynamics impact not only individuals’ health behaviors and the interpersonal and community relationships but also the health workforce, the health system, and the sociopolitical environment within which public health systems operate [32]. Regarding the latter, the politicization of health information also leads users to avoid critical thinking for selectively biased media content that can be amplified by platform algorithms [33-39].

### AI-Generated Misinformation

Comparatively, disinformation may be understood as a subset of misinformation, distinguished primarily by its intentionality, which often makes it more insidious and damaging to public trust [40]. Misinformation can be defined as follows [41]:

Misinformation is when false information is shared, but no harm is meant.

In the context of health-promoting behaviors and designed environments, understanding the continuum of elements in the information environment is essential to contextualize public health actions that aim to prevent harms to health and well-being [42]. This continuum starts with *questions* and *concerns*, the natural inquiries that people have during health crises. When these are not addressed, it can lead to *information voids* in which people search for answers but find none from credible sources. These information voids are situations in which people are especially on the lookout for health information, and when they fail to find it from credible, accurate sources, this creates fertile ground for exposure and susceptibility to *misinformation* (unintentional falsehoods) and *disinformation* (deliberate falsehoods), which can resonate with a person’s innate values or preexisting attitudes or perspectives. People may share information online because it is aligned with their beliefs, values, and experiences, which they want others to see, and therefore, these prevailing beliefs can snowball into prevailing narratives that can undermine trust in public health systems.

The rise of AI-generated media and content (eg, deep fakes, news articles, statistics, photos, and infographics) makes it increasingly difficult to understand health information objectively and its relevance and trustworthiness, ultimately complicating communication of health guidance, health risks, and benefits of health interventions and programs [43,44]. Reflecting this risk, there has been a rise in low-quality AI-generated content on the internet, which has been referred to as *digital sludge* or *the funkification of the internet*, adding to concerns that low-quality AI-generated content will be further incorporated into general-use commercial LLMs, thereby diminishing the quality of LLM outputs over time [45].

Generating digital sludge is a strategy used by health-harming industries in marketing their products, with the alcohol industry being just one example [46]. Finally, the concept of disinformation itself might need to be re-evaluated given the challenges of attributing intentionality in the context of AI-generated content that may be mediated by user-led prompts, impacted by model hallucination, influenced by underlying training data or AI guardrails (eg, responsible AI frameworks or lack thereof and curated knowledge bases), or facilitated by agentic AI models that focus on achieving goals versus outputs. Inherently, these features of GenAI technologies make it harder to discern intentionality without understanding the intent of the human users who use these tools for engaging in health communication and information dissemination.

In addition to being used for content creation, GenAI models are being built into many web portals, internet platforms, apps, and devices, therefore impacting the quality of the information to which individuals are exposed. However, these applications often prioritize popularity or relevance over accurate data sources, potentially reinforcing the imprecisions of the algorithm or of the user’s own preferences (which can be harmful, such as the promotion of ineffective treatments, amplifying health myths) as results are tailored to similarly situated users. An example of this challenge is the memory function of widely used GenAI platforms (eg, ChatGPT and Claude), which may skew a user’s information knowledge base over time without the user noticing [47]. This can lead users to reinforce inaccurate perceptions of health information and become more likely to share disinformation, which, over time, can lead to greater communal exposure to conflicting or false information that can erode trust in public health authorities and exacerbate existing societal divisions [23,43,44,48-50].

This emphasizes the need to understand how the integration of AI technologies into our information environment without appropriate public health safeguards can skew users’ perceptions and source accuracy, which demands a more nuanced approach to address not only the potential explicit harms of inaccurate narratives but also how the built information environment shapes people’s understanding of health and well-being.

To mitigate the potential impact of GenAI-generated misinformation and in the interpretation of the built information environment, we propose a comprehensive approach:

1. Technological solutions; development of advanced algorithms to detect and flag deep fakes and inaccurate information [48].
2. Public awareness; promoting critical thinking and digital literacy to recognize misinformation among the public.
3. Regulatory frameworks; implementing regulations to hold the full chain of dissemination of false information accountable (eg, the recently approved EU AI Act mandates disclosure when interacting with AI) [49].
4. Collaboration; engaging with technology companies, media, health care professionals, and governments to identify and mitigate the impact of misinformation [51].

### Societal Impacts

Mis- and disinformation are estimated to comprise 5% to 25% of the information environment and have wide-ranging impacts: psychological, physical, social, economic, and political [52-56]. Accordingly, these previously described information ecosystems—where sensationalist reporting, conflicting expert opinions, and slow issuance of health guidance create confusion among the public—undermine trust and enable misinformation exposure, endangering public health campaigns aimed at improving health outcomes [12,57,58]. For example, during a pandemic, this brings risks such as vaccine hesitancy and disregard for public health measures [58-60]. In addition, the health information system can lead to other behavior changes, with patients self-diagnosing more, requiring unnecessary and potentially harmful examinations and treatments, and possibly forgoing preventive or care options [61,62]. Finally, it is important to note that vulnerable communities (eg, those with low digital or health literacy or those who lack adequate access to health care services or coverage) are particularly susceptible to the deleterious impact of infodemics [63].

On the other hand, when combined with other strategies to strengthen public health surveillance, health promotion and education, and health informatic systems, GenAI has the potential to be an ally in the detection and mitigation of misinformation. For example, initiatives such as the health-related misinformation detection framework, SimSearchNet by Meta, and SynthID from Google DeepMind have the potential to act as agents for detecting health misinformation and AI-generated content, therefore being able to support the spread of accessible and accurate information [64-69]. In addition, GenAI may also be useful to understand geographical and temporal patterns of information and misinformation, analyze large datasets quickly for prevailing misinformation narratives that emerge from specific communities of interest, and better predict new trends and forecasts as health emergencies arise [70]. Furthermore, it has the potential through chatbots and other tools to enhance health literacy; accelerate information dissemination; support treatment adherence; enable early diagnosis; and contribute to disease surveillance, risk assessment, and mental health support if used appropriately and gated and guided by evidence-based information and ethical principles [71-74]. An example is the *digital health promoter prototype* Sarah, a GenAI chatbot and digital health promoter assistant developed by the World Health Organization (WHO) that provides guidance on healthy habits, mental health, noncommunicable diseases, and misinformation handling through online *face-to-face* conversations [75-82]. Examples of public health surveillance and health informatic systems include SIRVD-DL (Susceptible, Infected, Recovered, Vaccinated, and Deceased–Deep Learning) prediction model for COVID-19 surveillance, the WHO’s Global Influenza Surveillance and Response System, and AI for Pandemic and Epidemic Preparedness, which could benefit from integration with AI tools to tackle global health challenges, including in the context of identifying and contextualizing the impact of misinformation on health outcomes [83]. Other uses of GenAI aimed to support health behavior change have focused on improving literacy, access, and operational efficiency in projects spread across low- and middle-income countries in both Asia and Africa (Jacaranda Health, Viamo, Girl Effect, Audere, and Noora Health) [84]. However, the utility of GenAI tools to carry out shared public health objectives and goals lacks sufficient evidence and requires additional research. The overuse of such tools may lead to an overestimation of their actual value as current evidence does not yet support their widespread implementation. Nevertheless, many enterprises are already integrating GenAI functionalities into everyday activities.

While technological innovation can help shape healthier information environments, it is crucial to address these challenges holistically considering the broader, governance, regulatory, social, and informational contexts that can be informed by young professionals, who will arguably be most impacted by the implementation of these technologies in current and future public health and medical settings [85].

## Part 2: The Role of Public Health and Medical Professionals in LLMs and Information Ecosystems in Health

As discussed previously, the impact of the information ecosystem and mis- and disinformation on health and well-being in the GenAI era is vast and ever-evolving, with constantly new versions of LLM tools and systems that use them. Hence, it is crucial to take into account the views of young health professionals across various fields and regions to better understand current and future opportunities and challenges. We consulted medical doctors, public health professionals, and other health experts (researchers and psychologists) from 5 continents. The following sections present their perspectives through a unified response developed from the intersection of their individual blind answers.

### What Roles Do LLMs Currently Play in Your Professional Area, and How Can They Enhance the Propagation of Safe and Reliable Health Information?

LLMs are transforming health care by automating administrative tasks; enhancing clinical, public health, and administrative data management; and supporting health education and research. Examples of solutions under development that integrate LLMs include patient education, automated medical record writing, and providing suggestions for patient diagnosis and management [86]. In public health, LLMs can aid in data treatment, contributing to predictive analysis and response strategies during health emergencies and conducting rapid contextualized analysis of community sentiment and health behaviors to generate infodemic insights and inform adaptive delivery of public health responses, programs, and communications. Despite these advancements, the integration of LLMs into health systems and public health practices faces significant regulatory and ethical challenges. For example, Health New Zealand – Te Whatu Ora has specifically advised against the use of GenAI in health due to concerns over privacy, accuracy, and ethics. To address these issues, the WHO has called for stronger evidence on the design, training, and validation of AI-supported applications, yet global regulatory frameworks remain insufficient to oversee the full life cycle of AI in health care and public health [87].

### How Is the Integration of AI and LLMs Transforming Patient Communication and Overall Health Outcomes in Your Field?

AI and LLMs can transform patient-clinician communication not only in the consultation process but also by extending this communication beyond traditional consultation boundaries. These technologies can collect clinical information before appointments, handle administrative tasks such as record writing, and facilitate follow-up communications. If AI and LLMs can reduce the nonclinical workload of clinicians, this could allow health care providers to spend more time interacting with patients, thereby enhancing efficiency and quality of care. However, this productivity gain may only increase the number of patients seen without necessarily improving the quality of care.

In addition, if their current limitations and risks are overcome, GenAI tools have the potential to contribute to health promotion, risk communication, health education, and social behavior change efforts in public health systems. These technologies can personalize messaging, analyze real-time health data, and automate responses, enabling more targeted health education and behavior change interventions. AI can enhance risk communication by providing tailored, timely health warnings and support behavior change through internet-based counseling and interactive tools.

Furthermore, AI-powered tools are being tested to contribute to virtual counseling, adapted health information systems, and treatment adherence (eg, medication reminders) [88-90]. This can improve the patient-clinician communication and the care continuum, ensuring that patients receive comprehensive and continuous care. To facilitate this, such technologies could be integrated into previously developed digital solutions to make health care more accessible in rural areas, for example, through remote diagnostics and consultations in New Zealand and Brazil [91].

### How Do You Plan to Integrate LLMs Into Your Practice, and What Specific Applications Do You Foresee?

LLMs’ ability to process enormous amounts of data presents an opportunity to aid health care systems in managing patient outcomes. LLMs can transform previously unused data into actionable insights, enabling more effective management of patient outcomes. They can support clinical decision-making by providing clinicians with insights about patients in similar situations or generating concise patient summaries. If we can eliminate biases and other current limitations, LLMs can potentially identify patterns in patient data that may indicate a risk of developing certain conditions (digital twins), enabling early diagnosis and interventions, and remind patients and health professionals about regular checkups and screenings [92]. In this case, by analyzing and organizing large volumes of data, LLMs may facilitate regulated, accountable, and auditable data interoperability, increasing clinicians’ effectiveness and accelerating access to patient information when necessary.

Furthermore, LLMs can improve resource management. For example, in Germany, one of the authors is working on a project that is exploring the integration of LLMs into nonemergency urgent care to help with patient navigation—this system determines whether a patient needs an in-person urgent care appointment, a telemedical consultation, or over-the-counter medication from a pharmacy or can wait until normal business hours for their general practitioner.

### What Policies and Ethical Guidelines Do You Think Are Necessary to Responsibly Integrate GenAI Into Health Practices Worldwide (and Locally)?

The responsible integration of GenAI into health care requires robust global policies to avoid health risks, privacy issues, and biases (a study of a large US hospital database suggests that eliminating racial bias in triage algorithms would increase the percentage of Black patients who receive additional help from 17.7% to 46.5%) [93]. Thus, formulation of relevant policies and guidelines is urgently needed. Adherence to existing standards such as the General Data Protection Regulation (EU), HIPAA (Health Insurance Portability and Accountability Act; United States), and Lei Geral de Proteção de Dados (Brazil) is foundational. However, people living in countries without consumer protections are disproportionately affected by how commercial actors use their communities to develop, test, and deploy AI-supported tools, which may further exacerbate health and well-being inequities worldwide. Given the international nature of AI data markets, regional and national strategies should seek to align with global guidelines, with international organizations such as the WHO respecting the sovereignty of countries and the agency of individuals. Policies should balance innovation with patient safety and respect, ensuring practical implementation. The EU AI Act, for example, aims to create a comprehensive regulatory framework for AI, addressing risks and promoting ethical AI development and use within the EU.

Integration of these technologies must provide patients with easy options to opt out of data sharing without facing adverse consequences. In addition, specific consumer protection against deceptive marketing to vulnerable populations (eg, children), protection against hate speech, and protections of freedom of expression are essential. Furthermore, addressing the risk of propagating biases from training data is crucial as it impacts the fairness and accuracy of AI outputs. Ensuring inclusive and representative datasets is essential for equitable treatment of all patient groups. In addition, GenAI systems must be continuously validated in a transparent manner to increase data reliability, avoid issues related to copyright and intellectual property rights, and maintain trust and efficacy. In line with the Global Digital Compact, AI governance must be anchored in human rights and international law, with ethical guidelines, regular compliance reviews, and data security ensuring that these technologies benefit all [94-96].

### What Future Impacts of GenAI in Health Care and Public Health Are Underexplored or Underestimated, and How Should the Health Professional Community Prepare for These Changes?

The future impacts of GenAI in health care are promising but need to be further evaluated for key public health areas: disease surveillance, prediction of disease outbreaks, assessment of individual health risks, and suggestion of preventive measures. These advancements could lead to lower health care costs and better patient outcomes. In addition, GenAI has the potential to aid medical education through personalized learning experiences, virtual simulations, and real-time feedback. It can also streamline administrative tasks such as scheduling, billing, and resource allocation. Furthermore, in direct patient care, information from different centers remains highly fragmented worldwide. Patients often forget details about their medications, medical history, and previous treatments or surgeries. GenAI could help by securely integrating and condensing these data, reducing both missing and overlapping information, similar to what is being developed in the European Health Data Space.

While future health professionals will not need to be technology experts, they must effectively communicate with technology specialists. Therefore, every health degree should include GenAI training, including risks and limits, to prepare students for this integration. There will also be a growing need for professionals who can bridge management, technological, and health competencies to manage these complex systems, which can lead to work overload.

However, significant risks and challenges must be addressed. Public and ethical oversight, equity, and social participation are essential to prevent biases and ensure fair treatment for all patient groups and communities. Adequate resourcing of public communication; social participation; health promotion and health education capacities; and multilevel building of health, digital, information, and media literacies in communities and in the workforce is crucial. In addition, data sovereignty of peoples and nations (including indigenous communities experiencing inequities) is a crucial but underdeveloped area with important implications as AI progresses [97].

Policies must respect the data sovereignty of communities to ensure the ethical and equitable use of AI. These steps are necessary to foster acceptance and understanding of AI in health care.

## Part 3: Discussion and Call to Action

In the third part of this Viewpoint article, we focus on summarizing the potential impacts of GenAI in various areas of society, with a specific emphasis on education and training, research and innovation, and policy and ethics.

While we are not specifically prescriptive, allowing for contextualized implementation and locally defined metrics, we aim to inspire actionable advancements across these areas. We also highlight that networks such as the WFPHA are well positioned to support these efforts, whether through capacity building, literacy initiatives, or collaboration on the responsible integration of GenAI into public health.

### Education and Training

GenAI transformation is happening, and therefore, comprehensive training to future generations as well as to the current workforce is necessary. A commitment to maturation of AI literacy is necessary not only to improve the productivity of GenAI models and agents but also to ensure that consumers of GenAI-generated content are aware of its potential risks and biases and how to mitigate them [80]. In addition, health presents particular needs and serious implications for individual and community health, and therefore, specific continuous training for health and public health professionals should be prioritized.

Educational efforts must be comprehensive, addressing areas that continue to be debated in the GenAI space, including data privacy, policy implications, and algorithmic bias, while also promoting transparent and ethical design of GenAI systems and applications that are based on them. As previously discussed, promoting digital, media, and health literacy is central to infodemic resilience. This must be embedded into training programs for both the current workforce and future generations [29,54,98-103].

Therefore, we propose the following call to action: *increase health literacies (health, media, information, and science) and specifically AI literacy starting as early as preuniversity levels* to prepare future generations for the AI-driven health care landscape, as well as offering courses on AI use, risks, biases, and management of AI-generated information in all health degree programs. Similarly, we recommend integrating health topics into fields such as computer science, ethics, and technology governance.

### Research and Innovation

While we understand the importance of research frameworks that address AI interventions, such as CONSORT-AI (Consolidated Standards of Reporting Trials–Artificial Intelligence), SPIRIT-AI (Standard Protocol Items: Recommendations for Interventional Trials–Artificial Intelligence), and the WHO’s guidelines, we call attention to higher-level research needs that AI should support. Accordingly, we believe that AI research and innovation in health should prioritize three key areas: (1) ensuring universal and equitable access to accurate and high-quality health information (recognized by some organizations as a fundamental human right) by developing innovative strategies and digital solutions that promote informed decision-making and improve access to health care; (2) supporting preventive health programs using AI tools to enhance early detection, risk assessment, and health promotion efforts; and (3) combating polarization and bias in health information, ensuring that content delivered to diverse populations is trustworthy, inclusive, and culturally sensitive. Furthermore, while GenAI can significantly enhance research capabilities through advanced writing and data analysis, interdisciplinary collaboration is essential to prevent the spread of appealing yet inaccurate research results and misinterpretation or misrepresentation [104-106].

Therefore, we also introduce a call to action for *incorporation of AI and ethics experts within public health organizations* to ensure accurate and ethical AI application.

### Policy and Ethics

Effective GenAI adoption in health care and public health requires collaboration among patients, civil society, and policy makers to establish ethical frameworks, regulatory oversight, and effective data governance. The Global Digital Compact, annexed to the United Nations Pact for the Future, is the first global commitment to data governance—in addition to calling attention to the need to take local action by 2030, it incentivizes a global policy approach on AI governance and clarifies the need of this space to respect human rights and international law [96]. This urgent need for greater transparency and accountability is recognized by stakeholders from different backgrounds, from nongovernmental to corporate and philanthropic backgrounds (as exemplified by the Partnership on AI) [107-111].

A responsible transition depends on comprehensive yet flexible policy development (such as the EU AI Act), robust ethical guidelines (such as the WHO’s “Ethics and governance of artificial intelligence for health: Guidance on large multi-modal models”), and a strong commitment to ensure health equity [49,112]. These are vital to avoid biases in AI models and ensure that GenAI benefits are distributed fairly.

Regarding ethics, the WHO has outlined 6 ethical principles to guide AI development in health: protecting autonomy, promoting well-being and safety, ensuring transparency, fostering accountability, ensuring inclusiveness and equity, and promoting sustainability [112]. WHO ethics guidance will soon extend to provide help and capacity building to public health authorities to establish infodemic management practices, policies, and strategies [113]. These guidelines emphasize the importance of fairness, transparency, and accountability, addressing critical concerns such as data privacy, algorithmic bias, and health equity to prevent AI from deepening existing health disparities. Finally, the recent adoption of the WHO Pandemic Agreement, aimed to set a global standard for pandemic prevention, preparedness, and response, does not contain specific reference to infodemics or dis- or misinformation but does recognize the importance of trust and transparency in communications on the topic [114]. This further emphasizes the need for continued international cooperation and coordination on using GenAI as an objective tool to enhance health communication.

Therefore, we also make a call to action for *international collaboration* to develop AI-related legislation that ensures global standards and practices respecting regional and national autonomies, with clear definition of high-risk applications in medicine and public health, and the *development of equitable ethical and regulatory guidelines and codes* to guide all sectors in the responsible use of AI in health care and public health that prioritizes health equity and human rights.

## Conclusions

GenAI integration in health care and public health presents both significant opportunities and substantial risks, especially as it intersects with the complex dynamics of health information ecosystems. The main threats involve exacerbating information overload and disinformation and AI-generated misinformation exposure, all of which can negatively impact individual and collective health behaviors. However, through responsible, participatory, and evidence-based AI development, we can mitigate these risks and enhance the accuracy and accessibility of quality health information.

As emerging leaders in public health drawing on the experiences of professionals from diverse backgrounds across 5 continents, we recognize this dual nature of AI technologies. To harness GenAI’s potential responsibly, we must prioritize AI literacy among health professionals and the public, integrate AI education into health curricula, and establish robust ethical guidelines and regulatory frameworks that promote equity and protect patient privacy. By taking proactive steps in education, research, and policy, we can leverage GenAI to enhance global health while safeguarding against its risks.

## Abbreviations

AI: artificial intelligence
CONSORT-AI: Consolidated Standards of Reporting Trials–Artificial Intelligence
EU: European Union
GenAI: generative artificial intelligence
HIPAA: Health Insurance Portability and Accountability Act
LLM: large language model
SIRVD-DL: Susceptible, Infected, Recovered, Vaccinated, and Deceased–Deep Learning
SPIRIT-AI: Standard Protocol Items: Recommendations for Interventional Trials–Artificial Intelligence
WFPHA: World Federation of Public Health Associations
WHO: World Health Organization
